# Detection of an IL-6 Biomarker Using a GFET Platform Developed with a Facile Organic Solvent-Free Aptamer Immobilization Approach

**DOI:** 10.3390/s21041335

**Published:** 2021-02-13

**Authors:** Niazul I. Khan, Edward Song

**Affiliations:** 1 Department of Electrical and Computer Engineering, University of New Hampshire, Durham, NH 03824, USA; mk1125@wildcats.unh.edu; 2 Materials Science Program, University of New Hampshire, Durham, NH 03824, USA

**Keywords:** GFET, aptamer, immobilization, biosensor, IL-6, pyrene, microfluidic, lab-on-a-chip

## Abstract

Aptamer-immobilized graphene field-effect transistors (GFETs) have become a well-known detection platform in the field of biosensing with various biomarkers such as proteins, bacteria, virus, as well as chemicals. A conventional aptamer immobilization technique on graphene involves a two-step crosslinking process. In the first step, a pyrene derivative is anchored onto the surface of graphene and, in the second step, an amine-terminated aptamer is crosslinked to the pyrene backbone with EDC/NHS (1-ethyl-3-(3-dimethylaminopropyl) carbodiimide hydrochloride/N-hydroxysuccinimide) chemistry. However, this process often requires the use of organic solvents such as dimethyl formamide (DMF) or dimethyl sulfoxide (DMSO) which are typically polar aprotic solvents and hence dissolves both polar and nonpolar compounds. The use of such solvents can be especially problematic in the fabrication of lab-on-a-chip or point-of-care diagnostic platforms as they can attack vulnerable materials such as polymers, passivation layers and microfluidic tubing leading to device damage and fluid leakage. To remedy such challenges, in this work, we demonstrate the use of pyrene-tagged DNA aptamers (PTDA) for performing a one-step aptamer immobilization technique to implement a GFET-based biosensor for the detection of Interleukin-6 (IL-6) protein biomarker. In this approach, the aptamer terminal is pre-tagged with a pyrene group which becomes soluble in aqueous solution. This obviates the need for using organic solvents, thereby enhancing the device integrity. In addition, an external electric field is applied during the functionalization step to increase the efficiency of aptamer immobilization and hence improved coverage and density. The results from this work could potentially open up new avenues for the use of GFET-based BioMEMS platforms by broadening the choice of materials used for device fabrication and integration.

## 1. Introduction

In recent years, graphene-based field-effect transistors (GFETs) and their uses as sensing platforms have been greatly successful in developing various microfluidic and lab-on-a-chip-based biosensors [1,2]. Their effectiveness in the detection of various biomarkers can be attributed to the graphene’s high carrier mobility, sensitivity to molecules and 2-dimensional geometry resulting in ultra-sensitivity and easy integration capability [3,4,5]. A GFET works based on the modulation of the graphene channel conductance (i.e., the channel current) between the source and the drain upon the application of an external electric field through the gate electrode. This principle can be exploited as a method for sensing of biomolecules since charged molecules that are in close contact with graphene (within the Debye length) will cause a change in the electric field leading to a modulation in the drain-source current (I_DS_) of the GFET. A significant difference between GFETs and other conventional FETs is their ambipolar transfer (I_DS_-V_GS_) curve which results in a minimum channel current at the Dirac voltage also known as the charge neutrality point (V_CNP_). Any change in the electric field induced by the adsorption of the charged molecules at the graphene surface essentially causes a shift in the charge neutrality point enabling GFETs to be used for ultra-sensitive detection of the target biomolecules [4,6]. 

**Figure 1:**
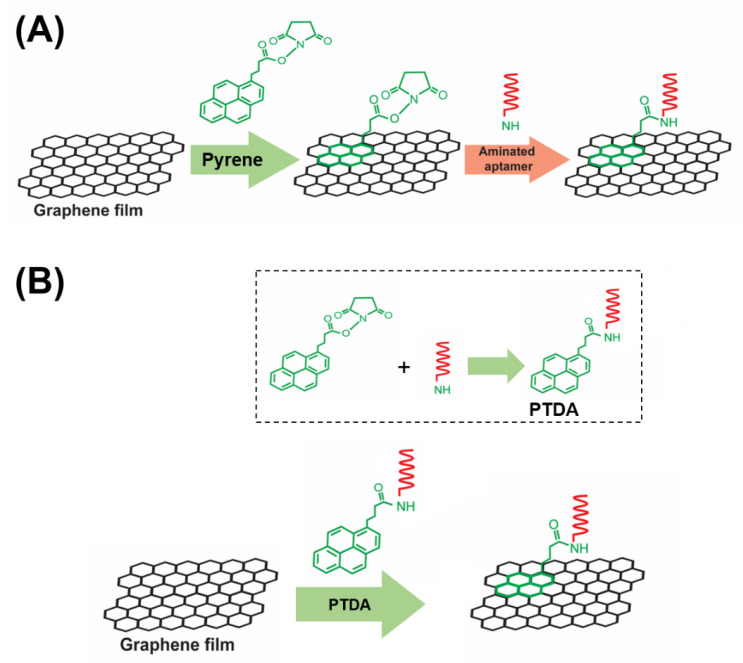
A schematic illustration of (**A**) a two-step aptamer functionalization on graphene requiring the use of organic solvents to dissolve and disperse PBASE (1-pyrenebutyric acid N-hydroxysuccinimide ester) and (**B**) the proposed one-step modification process of aptamer probes on graphene-based field-effect transistor (GFET). Inset: the crosslinking of PBASE with an aminated DNA aptamer to form a pyrene-tagged DNA aptamer (PTDA) probe.

To enhance the target selectivity in analyte detection, GFETs are commonly integrated with bioreceptors such as aptamers, antibodies and so forth. In this work, aptamers are used as representative target recognition probes. A major part of the implementation of such aptameric GFET devices is the reliable immobilization of aptamer probes onto the graphene channel of the device. In general, there are two main strategies for functionalizing aptamers on graphene, namely, the covalent and the non-covalent immobilization approaches. While the covalent approaches offer certain advantages over non-covalent methods in terms of stability and functionality, they unavoidably altar the physical properties of graphene. Hence, non-covalent modifications have been frequently used in order to maintain the inherent properties of a pristine graphene [7]. A typical non-covalent attachment of aptamers on graphene involves a two-step method as shown in Figure 1A. In the first step, a pyrene is anchored on the graphene via the π-π stacking interaction and in the second step, the amine-terminated aptamer is attached to the pyrene via the EDC/NHS (1-ethyl-3-(3-dimethylaminopropyl) carbodiimide hydrochloride/N-hydroxysuccinimide) crosslinking chemistry [8]. 

**Figure 2:**
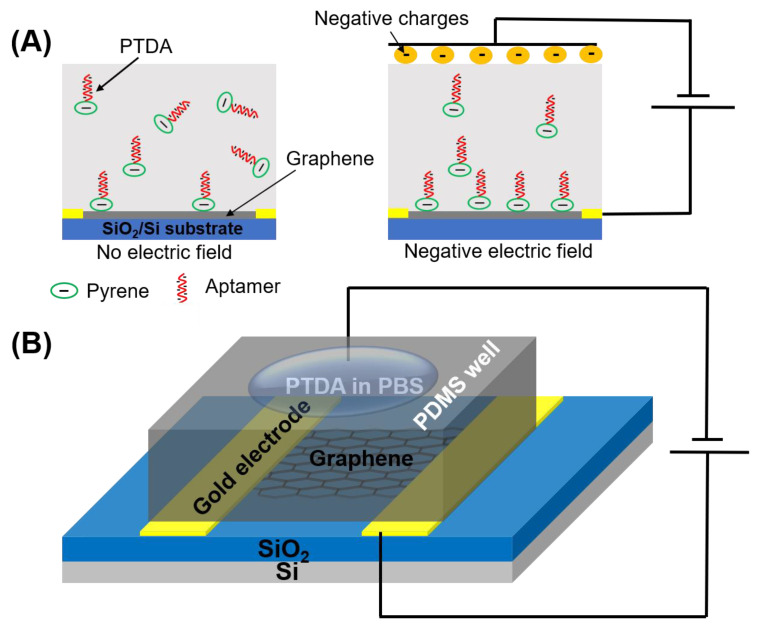
Schematic illustration showing the effects of applying an external electric field during the functionalization of pyrene-tagged DNA aptamers (PTDA). (**A**) The distribution of PTDAs in the incubation buffer without and with the external electric field and (**B**) the device setup for applying the external E-field during the PTDA functionalization on GFET.

A commonly used pyrene-based crosslinker, such as PBASE (1-pyrenebutyric acid N-hydroxysuccinimide ester) as shown in Figure 1, requires organic solvents such as Dimethyl formamide (DMF) or Dimethyl sulfoxide (DMSO) in order for it to be well-dispersed in a solution. However, such solutions, being strong polar aprotic solvents, can dissolve most organic compounds [9] and can become an issue for a number of applications especially in lab-on-a-chip and point-of-care diagnostic platforms [10,11,12]. Such devices often utilize thermo plastics, flexible polymers and passivation layers that are vulnerable to organic solvents resulting in fluid leakage and other irreversible damages. Therefore, to circumvent these challenges, an organic solvent-free aptamer immobilization method is highly desirable and would also allow more flexibility in choosing the materials in device fabrication. In this paper, we explore the feasibility of using an organic solvent-free aptamer functionalization technique where the amine-terminated DNA aptamers are pre-tagged with pyrene groups. These pyrene-tagged DNA aptamers (PTDA) are easily soluble in an aqueous buffer and can be anchored onto graphene surfaces (Figure 1B) without the need for organic solvents. Wu et al. reported a GFET-based biosensor for selective detection of *E. Coli* with the aid of pyrene-tagged DNA aptamers [13]. In their work, the pyrene was incorporated during the synthesis process of the aptamers and the purification of the aptamers was also conducted using column chromatography. Another GFET-based biosensor was developed by Farid et al. for the detection of tuberculosis biomarker IFN-γ using pyrene tagged aptamers [14]. However, the aptamers were dissolved in DMF for diluting and immobilization on GFET surface. Inspired by the previous developments, we propose in this work a cheaper and simpler method to pyrene conjugation of the aptamers. Although pyrene conjugation on the terminal of oligonucleotides has been well-established [15], the main novelty of this work is in the use of such pyrene-tagged aptamers in the development of GFET-based protein biosensor. Here, we demonstrate that GFET-based biosensors developed using aptamers pre-conjugated with pyrenes are also effective in protein biomarker (IL-6) detection. In our approach, the PTDAs were formed by crosslinking the pyrene groups to the commercially available amine-terminated aptamers. Furthermore, to separate out the unreacted pyrenes, a simple purification was performed by precipitation with the help of a centrifuge. Following the synthesis, the pyrene-tagged aptamers were anchored onto the graphene surface. Moreover, the efficiency of the aptamer immobilization was enhanced by applying an external electric field (E-field) to the GFET through the gate electrode immersed in the PTDA solution (Figure 2). Generally, in the absence of an external electric field, the amount of PTDAs anchored on the graphene surface is limited by the rates of diffusion and mass transfer (Figure 2A (left)). However, by applying a negative electric field, the PTDA molecules, which are negatively charged due to the combined effects of the electron-rich pyrenyl groups and the negatively charged DNA strands, are pushed toward the graphene surface where they interact with the graphene through the formation of π-π stacking interaction thereby enhancing the immobilization rate and the surface coverage (Figure 2A (right)). This one-step functionalization method also eliminates the need for additional washing steps and thus reduces the time required for device fabrication. To demonstrate the effectiveness of this technique in GFET-based biosensor implementation, the developed platform was used to detect interleukin-6 (IL-6) protein, a well-known cytokine and a biomarker for immune responses, as a representative target analyte [16,17]. 

## 2. Materials and Methods

### 2.1. Materials

**Figure 3:**
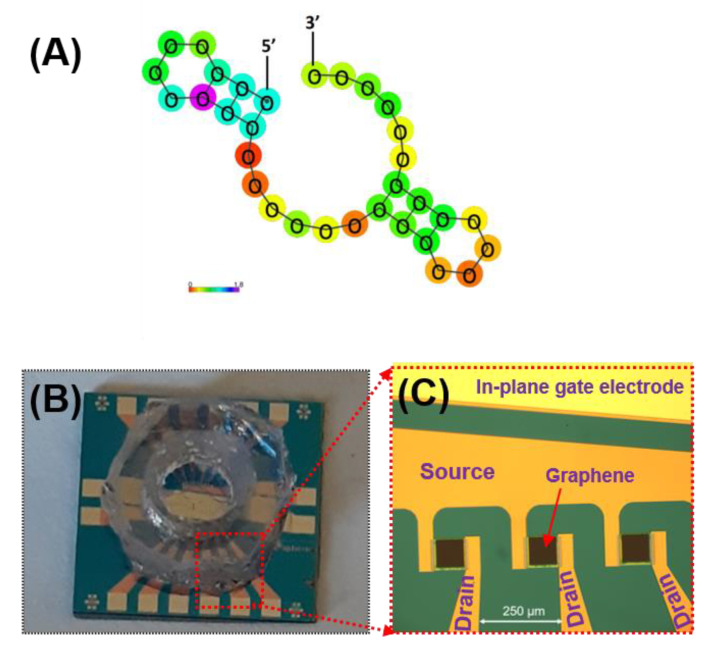
(**A**) Predicted secondary structure of the aptamer sequence purchase from Base Pair Biotechnologies, Inc. (Product #ATW0077) and the photographs of (**B**) the GFET chip (12 individual GFETs per chip) with PDMS well on top; and (**C**) the enlarged view of the 3 GFET devices of the chip (without the PDMS well). The source and drain electrodes are passivated with an insulating layer while the in-plane gate electrode is fully exposed.

The aminated aptamers having the specific affinity to mouse IL-6 (#ATW0077, K_D_ = 5.4 nM) and the resuspension buffer were purchased from Base Pair Biotechnologies, Inc. (Pearland, TX, USA). The aptamer’s affinity has been thoroughly characterized by the manufacturer and is shown to be specific toward IL-6 proteins. The predicted secondary structure of the aptamer sequence is presented in Figure 3A. PBASE (1-pyrenebutyric acid N-hydroxysuccinimide ester) was purchased from Santa Cruz Biotechnology (Dallas, TX, USA). The GFET chip (GFET-S20) fabricated on SiO_2_/Si was purchased from Graphenea, Inc. (San Sebastián, Spain). The gold electrodes (source/drain) surrounding the graphene channel were passivated with insulating layers consisting of Al_2_O_3_ (50 nm)/Si_3_N_4_ (100 nm). A polydimethylsiloxane (PDMS) well (3 mm diameter) was then integrated in-house to contain the liquid gate on the passivated area of the electrode as well as to reduce liquid evaporation during measurements and incubation steps. Figure 3B,C show the image of the GFET chip consisting of 12 individual GFET devices with a PDMS well placed over the sensing area. 

### 2.2. Formation of Pyrene-Tagged DNA Aptamer (PTDA) Probes 

The crosslinking of the pyrene groups to the aptamers to form PTDAs was achieved by incubating the aminated aptamers with PBASE dissolved in DMF following the protocol provided by the aptamer manufacturer [18]. Briefly, 50 µL of 100 µM IL-6 binding amine-linked (at the 5՛ end) aptamer in amine resuspension buffer was mixed with 1.26 µL of 10 mg/mL PBASE and incubated for 1 h in the dark. Then, 5 µL of 3 M sodium acetate was added to the aptamer/PBASE mixture followed by the addition of 125 µL cold ethanol (100%). The mixture was then placed in the freezer for 25 min followed by centrifugation at 13,000 RPM for 15 min causing a pellet formation as a precipitate. The precipitate was collected by decanting the supernatant and then washed with 70% ethanol followed by resuspension in 0.01× phosphate buffer saline (PBS). As per the manufacturer datasheet, the conjugation efficiency of the protocol varies from 50%–90%. Although the pellet is expected to contain a very high yield of conjugated PTDAs, it also includes some unconjugated aptamers or PBASE which can negatively impact the sensor performances. Also, there is the possibility of multi-conjugated aptamers due to the interaction with amine groups in the nucleobases. To obtain a precise yield of successful conjugation between aptamers and PBASE, a more complex analytical tools such as high-performance liquid chromatography (HPLC) could be used to experimentally investigate the conjugation efficiency.

**Figure A1:**
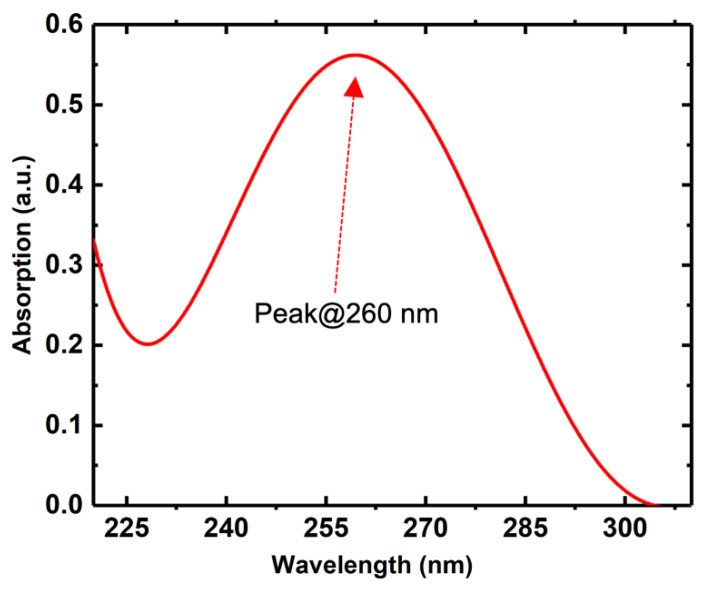
UV-Vis spectrum of the pyrene tagged DNA aptamer. The peak at 260 nm is the characteristic DNA peak that corresponds to the presence of DNA nucleobases in the synthesized product.

The concentration was determined by obtaining UV-Vis spectra measured with a Nanodrop spectrophotometer. Figure A1 (Appendix A) shows the UV-Visible spectrum of the resuspended PTDA. The peak at 260 nm corresponds to the presence of DNA nucleobases in the solution [19]. Although the UV-Vis spectrum is not able to provide the qualitative information about the aptamers (i.e., whether it is denatured or intact) we anticipated that the majority of the aptamers are in the properly functioning condition as evidenced by our GFET measurement results. Afterwards, the PTDA solution was stored at 4 °C. 

### 2.3. Immobilization of PTDA on Graphene 

For GFET functionalization and measurements, a PDMS well was constructed over graphene to avoid evaporation of liquid. The solution containing PTDAs (2 µM in 0.01× PBS) was loaded into the well and a negative electric field (−400 mV) was applied to the solution using a wire inserted into the PTDA solution for 4 h as shown in Figure 2B. Then, the GFET device was rinsed with DI water to remove any unbound PTDA probes.

### 2.4. Selective Detection of IL-6 Protein

After functionalizing the GFETs with IL-6 binding aptamers, various concentrations (0.1, 1, 10 and 100 nM) of IL-6 protein in 0.01× PBS with 2 mM MgCl_2_ were exposed to the sensing area (PDMS well) for 10 min each. To investigate the selectivity and specificity, 100 nM of lysozyme (Lys) protein was also exposed to the IL-6 aptamer modified GFET in the same buffer for the same duration. 

### 2.5. Electrical Measurements

For electrical measurements, the devices were placed on a probe station (Micromanipulator, 450 PM-B) and a Keysight B2902A source/measure unit (SMU) was used to measure the I_D_–V_GS_ transfer characteristics. Voltage control and data acquisition were performed using a LabVIEW program. During the measurement, the drain–source voltage (V_DS_) was biased at 100 mV and the drain current (I_D_) was read while the liquid–gate voltage (V_GS_) was linearly scanned from 0 V to 1 V with a voltage step of 12.5 mV using the in-plane gold gate electrode. A scan rate of 12.5 mV/s was maintained so that I_D_ is stabilized to ensure reliable measurements of the I_D_–V_GS_ transfer curves. 

## 3. Results and Discussion 

### 3.1. Characterization of Device Performances and the Effect of Gate Materials

#### 3.1.1. Mobility Calculation of the GFET Devices

**Figure 4:**
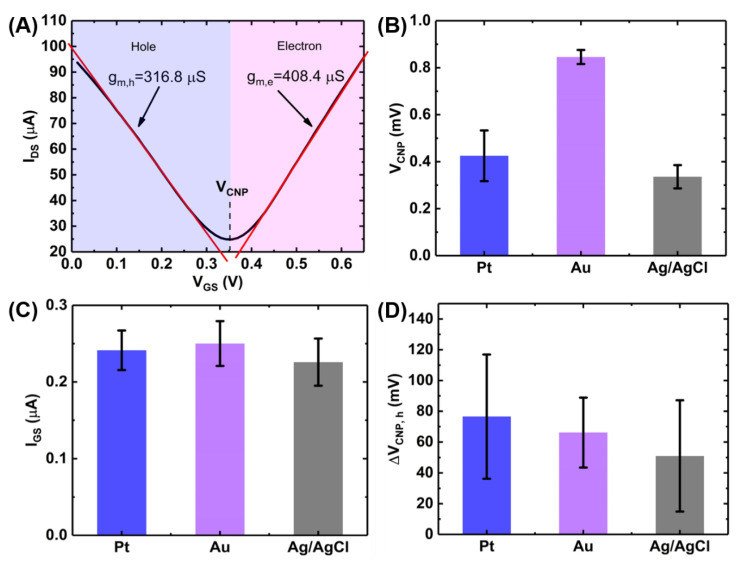
Characterization of GFET device performances and effect of gate materials. (**A**) GFET transfer curve showing the calculation of transconductance and bar charts showing the effect of gate materials on (**B**) V_CNP,_ (**C**) leakage current (rms value) and (**D**) gate hysteresis. The bar chart represents 1 standard deviation among measurements on 6 GFET devices on a single chip.

The transfer characteristics of a transistor in a linear region can be described as follows [20]:

$$I_{DS}=\frac{W}{L}.C_{TG}.\mu .\;\left(V_{GS}-V_{CNP}\right).V_{DS},$$

where, $\frac{W}{L}$ is the width-to-length ratio of the GFET channel, $C_{TG}$ is the total gate capacitance of the liquid gate, μ is the carrier mobility. Figure 4A shows the ambipolar transfer characteristics of a GFET resulting in a V-shaped curve where the left branch represents the increasing density of positive charge carriers (holes) and the right branch represents the increasing density of negative charge carriers (electrons) [21]. The critical transition voltage between the two regions where the current reaches a minimum is called the charge neutrality point (V_CNP_) or the Dirac voltage (V_Dirac_) [22]. The slope of the transfer curve ($\frac{dI_{DS}}{dV_{GS}})$ in each region indicates the transconductance ($g_{m})$ for the hole and the electron, respectively and can be calculated by measuring the slopes of each branch of Figure 4A. Mathematically, $g_{m}$ can be expressed as [23]:

$$g_{m}=\frac{dI_{DS}}{dV_{GS}}=\frac{W}{L}.\;C_{TG}.\mu .\;V_{DS}\;,$$

which leads to the following expression for mobility:

$$\mu =\frac{L}{W}.\frac{g_{m}}{C_{TG}.V_{DS}}.$$

Therefore, the average carrier mobilities for holes and electrons were calculated to be: $\mu_{h}=1920\pm 61$ cm^2^/V/s and $\mu_{e}=2475\pm 163$ cm^2^/V/s, where $V_{DS}=100$ mV and the value of $C_{TG}$ was taken to be 1.65 µF/cm^2^ [13]. This high values of the carrier mobilities indicate the suitability of the GFETs for sensing applications.

Another important parameter that affects the sensitivity of the GFET devices is the on-off ratio (I_ON_/I_OFF_) of the drain-source current. The larger the value of the on-off ratio, the better the sensitivity of the GFET since the device will exhibit better immunity to noise. An on-off ratio value of ~8 was calculated for the used devices which is above average for GFET devices grown by a CVD technique [22]. 

#### 3.1.2. The Effects of the Gate Material on Device Characteristics

We also investigated the effects of different gate materials (Pt, Au and Ag/AgCl) on the GFET transfer characteristics illustrated in Figure 4B–D. Figure 4B shows the effects of the three gates on the Dirac voltage or the charge neutrality point (V_CNP_) for the 6 GFET devices on a single chip. As seen in the figure, Ag/AgCl gate electrode gives the lowest V_CNP_ among the three gate electrodes. However, the gold electrode provides the lowest device-to-device variations among the devices on a single chip. 

Figure 4C shows the effects of the gate material on the gate leakage current where each bar represents the RMS value of the leakage current (I_GS_) calculated from the I_GS_-V_GS_ curves (See Appendix B). The RMS value was calculated using the following equation:

$$I_{GS}=\sqrt{\frac{1}{n}}\sum_{i}i_{GS}^{2},$$

where n is the number of measurement points and $i_{GS}$ is the leakage current for each individual gate voltage. 

Gate leakage has been a very common phenomenon in liquid-gated GFETs and is primarily caused by the electrochemical redox reaction at the graphene/liquid interface resulting in an increased current flow which negatively impacts the sensing performances of the sensor. Though passivation of the exposed electrodes can reduce the leakage current, carbon clusters and photoresist residues during the wet transfer of CVD-graphene can act as a source of carbon leading to redox current during the device operation [24,25]. Among the three gate materials tested, Ag/AgCl resulted in the lowest gate leakage. 

Figure 4D shows the effects of the gate material on the hysteresis in the GFET transfer characteristics. Gate hysteresis or simply hysteresis, in GFET is the deviation of drain-source current upon reversal of the gate voltage sweep direction [26,27,28]. This causes a shift in the charge neutrality points in the forward and backward scans (See Appendix B). This shift (ΔV_CNP,h_) has been plotted in Figure 4D for the three gate materials. It can be seen that Ag/AgCl gives the lowest hysteresis.

Although Ag/AgCl demonstrates the best performance in terms of the operating voltage, gate leakage and gate hysteresis, in-plane gold electrode was used throughout the experiments as it gives the highest uniformity of the charge neutrality point among the devices on a single chip. Moreover, the in-plane configuration of the gold electrode which can be fabricated at the same lithography step as the source and drain electrodes enhances the compactness of the setup and allows the potential integration with the microfluidic platform [8]. 

### 3.2. Characterization of the Aptamer-Functionalized GFET Devices 

**Figure 5:**
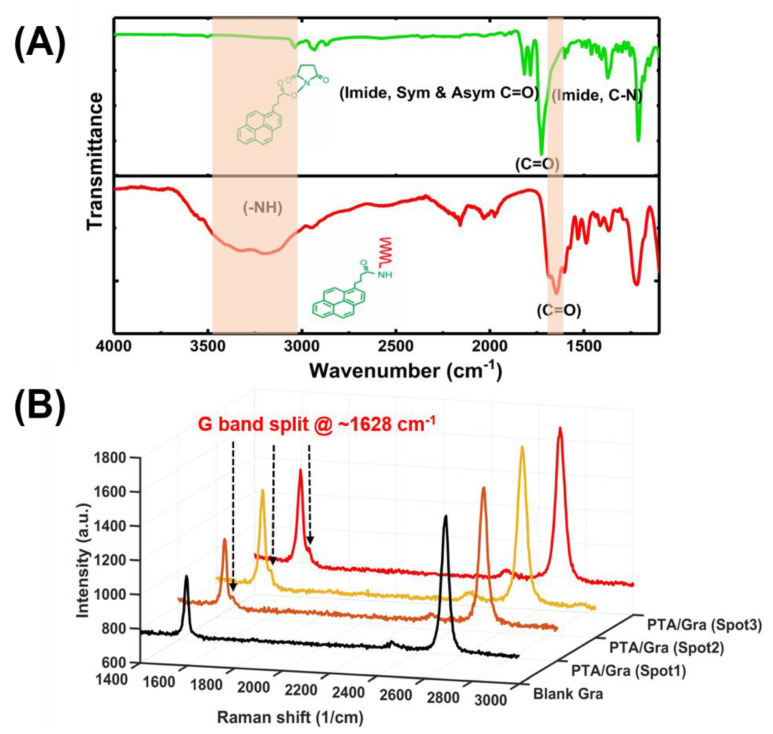
Optical characterization of the one-step functionalization of aptamer probes on graphene. (**A**) FTIR characterization of the amide bond of PTDA in dry state and (**B**) Raman spectrum (excitation by 532 nm) of three spots of a PTDA functionalized graphene along with that of blank graphene (Gra).

The synthesized PTDAs were first characterized to verify the presence of an amide bond between the PBASE and the aminated aptamer. The amide bond was characterized using the Fourier-transform infrared (FTIR) spectroscopy as presented in Figure 5A, where the presence of a strong peak at 1653 cm^−1^ (C=O stretching in the amide I) and the broad stretching vibration peak around 3300–3550 cm^−1^ (N–H from the amide, –OH group at the 3ʹ end of the DNA) confirm the amide bond formation [5]. Although amine groups from adenine, cytosine, guanine can also react with the NHS ester of the PBASE linker resulting in amide bond, this efficiency of these reactions are quite low compared with that with the primary amine group connected at the 5ʹ end of the DNA aptamer. Hence, it is expected that the amide peak at the FTIR is primarily attributed to the amide bond at the 5ʹ end of the aptamer.

After the amide bond was confirmed by the FTIR spectroscopy, the PTDAs were immobilized on bare graphene. The presence of the PTDAs on graphene was confirmed by Raman spectroscopy. For Raman measurements, two samples were prepared, a blank graphene and a graphene functionalized with PTDAs. The Raman spectra for the PTDA-modified graphene were taken at 3 different spots on the surface. Figure 5B shows the Raman spectra of the blank and the PTDA-functionalized graphene where the G-band split at around 1628 cm^−1^ indicates the anchoring of the PTDAs by π-π stacking interaction between the pyrene ring of the PTDA and the basal plane of graphene [8].

**Figure 6:**
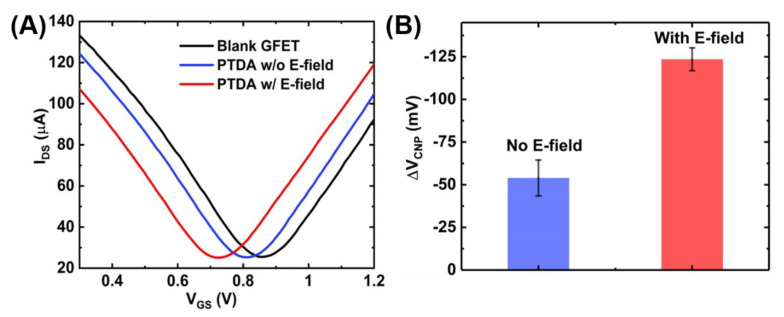
GFET transfer curves showing the effect of external electric field on functionalization of PTDA on GFET devices. (**A**) GFET transfer curves and (**B**) the corresponding shift in V_CNP_ (n = 5).

The anchoring of the PTDAs on graphene surface was also verified electrically by measuring the GFET transfer curves before and after the probe attachment. As shown in Figure 6A, the charge neutrality point shifts left upon PTDA immobilization on graphene. This negative shift is in accordance with the negative charges on the DNA backbone and the electron-rich pyrene group of the PTDA and is also consistent with the previous work by Wu et al. [13]. Furthermore, the effects of an external electric field were also characterized using the I_DS_-V_GS_ curves. Figure 6A further shows that when a negative potential is applied at the gate, the PTDA immobilization efficiency is enhanced which is reflected by the increased amount of the negative shift in the charge neutrality point (V_CNP_) compared to the case without the external electric field. With a negative potential at the gate, the negatively charged single-stranded PTDAs tend to migrate towards the graphene surface due to the electrostatic repulsion resulting in an increased density of the immobilized PTDA probes [16]. The bar graph in Figure 6B indicates an increased amount of shift in the V_CNP_ with respect to the blank GFET device for the two cases. With the electric field applied, the negative shift in the charge neutrality point was measured to be 123.53 mV which is approximately 2.5 times larger than that without the electric field.

### 3.3. Sensitivity and Selectivity Study for the IL-6 Detection

**Figure 7:**
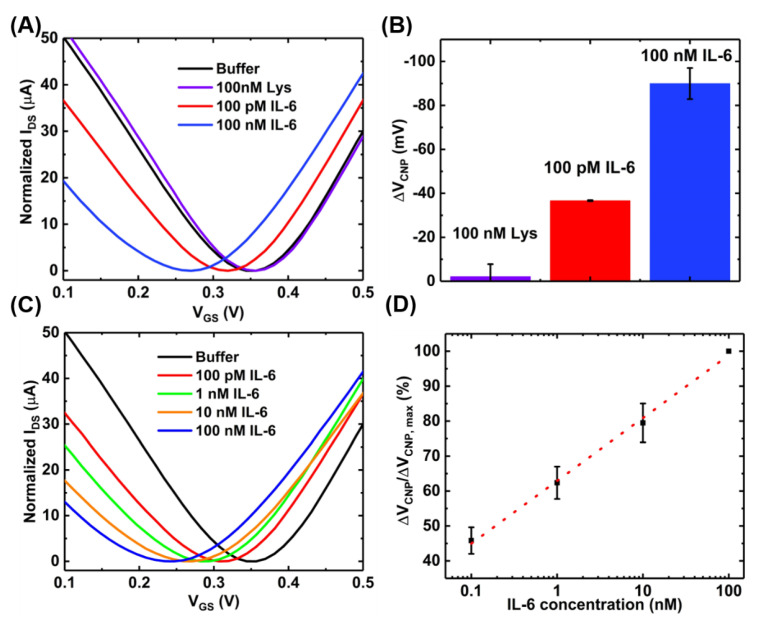
Sensing experiments with the GFET-based aptasensor at pH~7.4: (**A**) the transfer curves; (**B**) the bar chart showing the selectivity of the sensor (error bar with n = 3); (**C**) transfer curves of the GFET sensor when exposed to varying concentrations of IL-6; and (**D**) the concentration-dependent calibration curve (n = 3).

After the successful functionalization of the GFET with the PTDAs, the device was exposed to IL-6 proteins to characterize its sensing performances. Prior to IL-6 exposure, in order to test the sensor’s selectivity to its target, the GFET-based IL-6 sensor was exposed to 100 nM of lysozyme protein (a model interfering species) in 0.01× PBS with 2 mM MgCl_2_ for 10 min. Once the selectivity of the IL-6 binding aptamers was confirmed, the sensor was then exposed to various concentrations (100 pM, 1 nM, 10 nM and 100 nM) of the target protein (IL-6) in the same buffer and for the same exposure time. Figure 7A,B show the transfer curves and the bar graph, respectively, for each sample exposure. These results indicate that our sensor platform is minimally responsive to a non-target species (lysozyme) even when a relatively large concentration (100 nM) is exposed. By contrast, upon introducing 100 pM of the target biomarker IL-6 to the GFET sensing area, the charge neutrality point shifts to the negative direction by a significant amount indicating the specific analyte recognition by the aptamers as well as the target selectivity of the developed IL-6 biosensor. The charge neutrality point continues to shift to the left with increasing concentrations of IL-6 (Figure 7C). This consistent negative shift can be attributed to the n-type doping of the graphene channel by the bound IL-6 proteins which have an isoelectric point of 4~5.3 and therefore, is negatively charged under the buffer (pH = 7.4) used in the experiment [29,30]. The basis for this target-induced doping of graphene is on the target-induced conformational changes in the aptamers. In the absence of the target analytes, the aptamers anchored onto the graphene surface are in an unfolded, looped and flexible state. Upon exposure to IL-6, the target-induced conformational changes of the aptamers lead to a compact and stable shape. These structural changes in aptamers bring the negatively charged IL-6 protein to the close proximity of the graphene surface, possibly resulting in a direct transfer (doping) of electrons from IL-6 to graphene due to the π-π stacking interactions between the aromatic amino acids in IL-6 and the basal plane of graphene [29].

**Figure A3:**
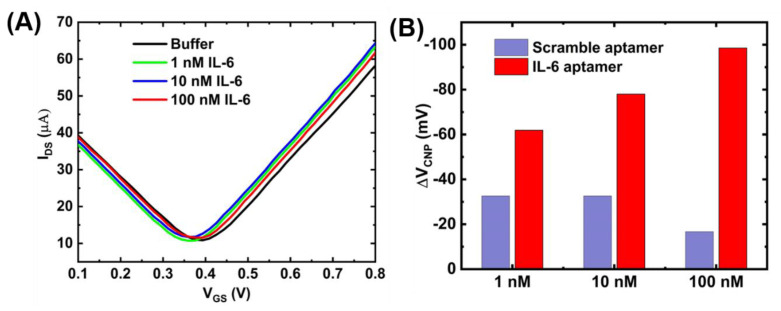
Scramble aptamer test: (**A**) GFET measurements of the scramble aptamer modified GFET upon exposure of different concentrations of IL-6 protein and (**B**) Comparative bar chart showing the response of IL-6 protein to the aptamer-modified GFET.

Also, the specificity of sensor was examined by functionalizing the GFET with a random sequence aptamer using the same protocol as the IL-6 aptamer and exposing different concentrations of IL-6 protein. The results are presented in Figure A3 (Appendix D) which shows negligible shift in the charge neutrality point after exposure to IL-6 protein. This further verifies that the IL-6 binding aptamer used in our sensor development exhibits specific target binding toward the IL-6 biomarker.

**Figure 8:**
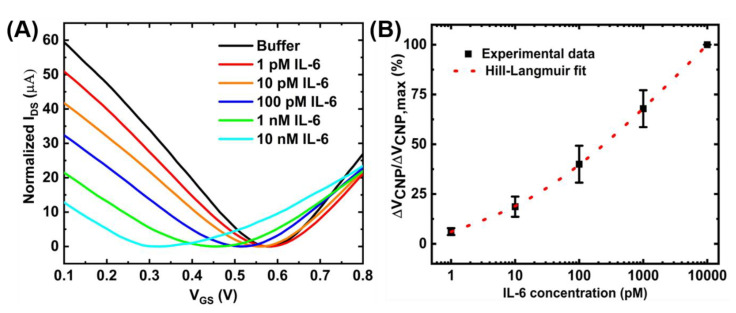
Detection of IL-6 with the GFET-based biosensor under the pH of ~3.64: (**A**) I_DS_-V_GS_ transfer curves for different concentrations of IL-6 protein and (**B**) the corresponding concentration-dependent calibration curve. The sample set is n=3 and error bar represents 1 standard error.

Figure 7D shows the calibration curve for a range of IL-6 concentrations obtained with a sample size of n = 3. The device-to-device variations were addressed by normalizing the sensor response (ΔV_CNP_) using the formula ΔV_CNP_/ΔV_CNP,max_, where V_CNP,max_ is the charge neutrality point corresponding to the maximum IL-6 concentration tested. With this detection method, the limit of detection of 100 pM was achieved. Although this detection limit is relatively higher than that achieved by the standard two-step immobilization of aptamers [16], the benefits of the proposed device fabrication method are easy and rapid immobilization of the aptamers. However, the sensing performances could be further improved by optimizing the device preparation steps. For example, increasing the number of washings steps during the purification stage of the PTDA synthesis process and optimizing the incubation time may lead to increased sensitivity. Moreover, adjusting the buffer pH to make IL-6 positively charged may result in an increased affinity between the positively charged IL-6 and negatively charged aptamer, possibly leading to enhanced sensitivity [31]. As an example, Figure 8 shows the IL-6 sensing result and the corresponding calibration curve of the GFET-based biosensor in the same buffer (0.01× PBS + 2 mM MgCl_2_) but with the pH adjusted to ~3.64.

The limit of detection (LOD) of the GFET-based IL-6 sensor under this pH environment was calculated to be ~8 pM which is an order of magnitude larger than that under the physiological pH (pH ~7.4). Figure 8B also shows the Hill-Langmuir fit (See Appendix C) of the experimental data [8]. The sensing performances of the proposed sensor are comparable to other results published in the literature. For example, Hao et al. have achieved a detection limit of 1.22 pM and a detection range of 1 pM–1 nM using conventional aptamer immobilization methods. From the Hill-Langmuir equation, the dissociation constant K_D_ is estimated to be 3.4 nM, similar to the value (5.4 nM) reported by the manufacturer for the aptamer-target pair. 

## 4. Conclusions

We have developed a facile and rapid immobilization technique to attach target recognition probes on the GFET-based biosensing platform. The developed sensor was able to selectively measure IL-6 protein biomarker with the detection limit in the picomolar range. The sensitivity can be further improved by increasing the incubation time, purification steps as well as by adjusting the buffer pH to an acidic region. The proposed organic solvent-free aptamer immobilization technique is not only polymer friendly (and therefore allows more flexibility in device design and fabrication) but also simplifies and shortens the graphene modification process by eliminating the extra step needed for anchoring the linker molecules and the subsequent washing steps. We have also demonstrated that an external electric field can be used to enhance the efficiency (~2.5 times) of the aptamer immobilization on the graphene surface. Our proposed technology has the potential to be used in monitoring IL-6 from real physiologically relevant fluid samples such as sweat, serum and cerebrospinal fluid.

## Figures and Tables

**Figure A2:**
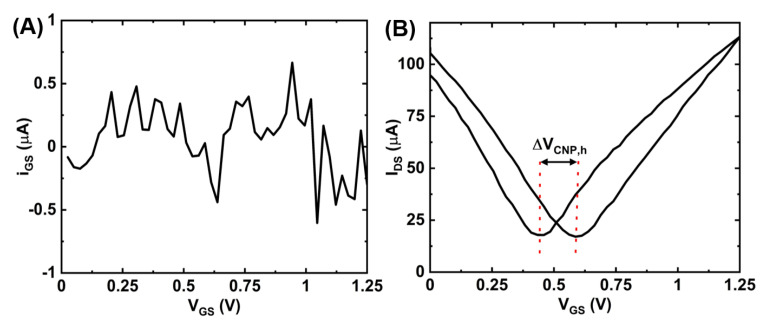
Measurement data for a gate (Pt) electrode: (**A**) gate leakage (I_GS_) vs. gate-source voltage (V_GS_) and (**B**) I_DS_-V_GS_ transfer curve showing the amount of hysteresis (ΔV_CNP,h_).

## Data Availability

All data collected in this study are presented within this article and the appendices.

## Appendix A

A1. Confirmation of the presence of DNA nucleobases in the synthesized product.

## Appendix B

A2. Leakage current and hysteresis in the GFET transfer curve.

## Appendix C

A3. Hill-Langmuir fitting of aptamer-protein equilibrium binding.

The calibration curve profile presented in Figure 8 can be best modeled by the Hill-Langmuir equation that describes the equilibrium binding of a ligand by a receptor [32,33,34]:

$$r=\frac{r_{0}+r_{m}{\left(\frac{c}{K_{D}}\right)}^{n}}{1+{\left(\frac{c}{K_{D}}\right)}^{n}}$$

where $r_{0}$ represents the estimated minimum response while all the binding sites are empty, $r_{m}$ is the estimated maximum response while the binding sites are occupied, c indicates the target concentration, $K_{D}$ is the effective dissociation constant which is defined as the concentration where half of the available binding sites are occupied and n represents the Hill coefficient.

Table A1 summarizes the values of the parameters that result in the best fit (R^2^ = 0.9925) for the Hill-Langmuir model of the calibration curve. A Hill coefficient value of *n* = 0.3 (which should be close to 1 under ideal conditions) indicates a decreased binding affinity with the target which may be caused by the interactions among the neighboring proteins or by the increased charge carrier scattering as more ligand bindings occur on the graphene surface [34,35]. The best fit value of K_D_ = 3.4 ± 2 nM is nearly identical to the value reported by the aptamer manufacturer. Based on the obtained calibration curve, the limit of detection (LOD) of our sensor is calculated to be ~8 pM.

**Table A1:** Summary of the Hill-Langmuir fitting parameters of the calibration curve.

**Hill-Langmuir parameters**	**Value**	**Error**
$r_{0}$	−9.6%	±3.1%
$r_{m}$	179.4%	±18.6 mV
$K_{D}$	3.4 nM	±2 nM
n	0.3	±0.03

## Appendix D

A4. Specificity test with a random sequence aptamer.

To test the specificity of the GFET sensor, the GFET was functionalized with a randomized aptamer sequence using the same one-step functionalization protocol as the IL-6 aptamer. The sequence of the randomized single-stranded DNA was: ATCAGGGCTAAAGAGTGCAGAGTTACTTAG. Following functionalization, the aptamer modified GFET was exposed to different concentrations (1, 10 and 100 nM) of IL-6 protein. The results are presented in Figure A3, which shows no significant shift of the charge neutrality point upon exposure of the IL-6 protein. This is due to the fact that the random sequence aptamer does not exhibit high affinity toward IL-6 protein suggesting the specificity of our sensor toward the target protein IL-6.
